# Role of Immunotherapy in the Treatment of Triple-Negative Breast Cancer: A Literature Review

**DOI:** 10.7759/cureus.31729

**Published:** 2022-11-21

**Authors:** Khushbu K Patel, Danial Hassan, Shaalina Nair, Sreedevi Tejovath, Simranjit S Kahlon, Aishwarya Peddemul, Rabia Sikandar, Jihan A Mostafa

**Affiliations:** 1 Internal Medicine, California Institute of Behavioral Neurosciences & Psychology, Fairfield, USA; 2 Health Care Profession, Ministry of Public Health, Doha, QAT; 3 Cardiology, California Institute of Behavioral Neurosciences & Psychology, Fairfield, USA; 4 Obstetrics and Gynecology, California Institute of Behavioral Neurosciences & Psychology, Fairfield, USA; 5 Psychiatry, Professional Psychotherapy, Cognitive Behavioral Psychotherapy, California Institute of Behavioral Neurosciences & Psychology, Fairfield, USA

**Keywords:** immune checkpoint inhibitor, tumor immune microenvironment, breast cancer, metastatic triple-negative breast cancer, cancer-immunotherapy

## Abstract

Numerous malignancies, including metastatic triple-negative breast cancer (TNBC), which has long been associated with a poor prognosis, have been transformed by the widespread use of immunotherapy. Immune checkpoint inhibitors (ICIs) that target and block programmed cell death-1 (PD-1) and programmed cell death ligand-1 (PD-L1) have demonstrated encouraging outcomes in the treatment of patients with metastatic TNBC. The PD-1 inhibitor pembrolizumab is the first-line treatment of metastatic PD-L1+ TNBC in combination with chemotherapy, and the PD-L1 inhibitor atezolizumab has also shown clinical activity. The median progression-free survival for pembrolizumab or atezolizumab combined with chemotherapy increased by 4.1 months and 2.5 months, respectively, with the addition of immunotherapy. Despite this progress, there is still more to be desired. The addition of immunotherapy to chemotherapy improved the pathological complete response (PCR) rate compared to chemotherapy with placebo in landmark phase III trials in the early-stage neoadjuvant context, whereas others reported no meaningful improvement in PCR. There are various ongoing trials that show that more research and studies are needed for components in the TNBC microenvironment and to further explore its importance in the treatment of TNBC.

## Introduction and background

A total of 6,85,000 people worldwide died in 2020 and 2.3 million women were diagnosed with breast cancer. Breast cancer is the most common cancer worldwide and in the United States with 29.6 percent of all diagnoses as of the end of 2020 when there were 7.8 million women still alive who had received a diagnosis during the previous five years [1,2]. Triple-negative breast cancer (TNBC) is an aggressive form of breast cancer that lacks the estrogen and progesterone hormone receptors and the human skin growth factor 2 (Her2) amplification. It makes up approximately 15-20 percent of all breast cancers [3-5]. It is made up of an immunological tumor microenvironment (TME) with high production of vascular endothelial growth factors (VEGF), tumor-infiltrating lymphocytes (TILs), tumor-associated macrophages (TAMs), extracellular matrix (ECM), connective tissue, stromal fibroblasts, and cytokines that stimulate the growth and migration of tumor cells, this TME plays a dual function in the development, progression, and metastasis of tumor [6-9].

The ECM is a key element of the TME. It is characterized by a network of different macromolecules that, in both healthy and sick states, forms the structural environment that determines the mechanical properties of the tissue [10]. Fibular collagen, fibronectin, elastin, and laminins are the predominant ECM molecules seen in solid tumors such as breast cancer [11,12]. Increased ECM deposition in breast and other solid cancers has been linked to poor survival in patients [13-19]. Tumor-associated collagen signatures (TACS) and alterations in collagen fiber architecture are suggestive of poor patient outcomes across all subtypes of breast cancer, including TNBC [14,18,20]. TILs are crucial in the killing and removal of tumor cells, but high expression of immune checkpoints inhibits immune cell function and promotes tumor immune escape [21-25], which excludes immune cell infiltration and also promotes immunosuppression and resistance to immune checkpoint inhibitors (ICI) [26-28]. These alterations in fibrotic ECM lead to cell behavior towards tumor progression [7,29,30], and the increase in tumor antigen expression provides the basis for immune system recognition and tumor removal. In order to diagnose and treat TNBC, it is crucial to comprehend the immunological microenvironment of the disease [31].

Chemotherapy is the backbone of treatment for both early-stage and metastatic TNBC because this tumor lacks hormone receptors and Her2 amplification, making endocrine therapy or Her2-directed therapy inefficient as targeted therapy [3]. Chemotherapy also lowers the risk of recurrence and death and is now frequently advised as preoperative (neoadjuvant) therapy [3]. Some patients experience a pathological complete response (PCR), while others do not, and their five-year overall survival rates (OS) range from 50 to 62 percent [32,33]. To improve patient survival, new immunotherapies, antibody-drug conjugates (ADCs), and other tumor- and stromal-targeted medicines must be developed for TNBC patients [34].

The immune system is involved in tumor development and progression and cancer cell detection and destruction. The antitumor immune response slows tumor formation and progression through tumor-directed immune responses involving cytolytic T cells [35,36]. Tumors must dodge the cytotoxic antitumor response through a variety of methods to progress. Immune evasion can take many different forms, including chronic activation of humoral immunity, invasion by T helper 2 (Th2) cells, innate inflammatory cells that are protumor-polarized, varying levels of tumor-specific antigens, negative immune checkpoints on tumor cells, and the absence of major histocompatibility complexes (MHC) on the surface of tumor cells [35,36]. Finally, these pathways work together to modulate anticancer responses while promoting tumor growth and disease progression. Early TNBC is still typically treated with a combination of several chemotherapeutic medications, most frequently given preoperatively to evaluate tumor sensitivity and modify postoperative systemic therapy as necessary [37]. In fact, individuals who have untreated residual illness after neoadjuvant chemotherapy are most at risk for recurrence [32] and benefit greatly from adjuvant capecitabine [38].

In this review, we will discuss the various modes of treatment for TNBC such as immunotherapy which includes ICI, the role of poly (ADP-ribose) polymerase inhibitors (PARPi), various clinical trials which are ongoing, completed, and active, not recruiting, etc., also see in a brief about role of vaccines in immunity to get a clear picture of what mode of treatment is followed and what is beneficial for patients in improving their prognosis.

## Review

Discussion

Immunotherapy

Immunotherapy is stimulation of the one’s immune system by either active immunization with cancer vaccines or passive immunization through tumor-specific antibodies and immune modulators, like immune-checkpoint inhibitors.

Immune checkpoint inhibitors: Immune checkpoints are a broad group of adaptive immune system regulatory points that play roles in self-tolerance and antitumor immunity. Physiologically, these checkpoints regulate the immune response negatively or positively, coordinating the intensity and type of reaction. Antibodies targeting cytotoxic T lymphocyte-associated antigen 4 (CTLA-4) and the programmed cell death protein 1 pathway (PD-1/PD-L1) have been the focus of many clinical trials using immune-checkpoint blockade. CTLA-4 and PD-1 in the tumor microenvironment act as negative regulators of immune activation, preventing anticancer immune response. Monoclonal antibodies work by eliminating suppression of the antitumor immune response [39]. Tumor antigens cause overexpression of several inhibitory receptors by T cells when a person develops a malignancy, which can lead to poor tumor identification [40]. Tumor cells secrete PD-L1, which interacts with PD-1 receptors on T cells and hinders the immune system's ability to recognize developing tumor cells [41]. Drugs like pembrolizumab, Nivolumab, and Atezolizumab target PD-1 receptors present in T cells or in tumor cells, which boosts antitumor immunity [42]. The PD-1/PD-L1 axis is activated when PD-1 binds to its ligand (PD-L1), inhibiting the immune response [43]. T cells, B cells, natural killer cells, dendritic cells, and macrophages are among the immune cells that are inhibited by PD-L1 expression in the innate and adaptive immune systems [44]. This high degree of PD-L1 expression in TNBC makes ICIs, such as anti-PD-1 treatments, easy targets [43-45].

PD-1 Inhibitors

Pembrolizumab: In the phase Ib KEYNOTE-012 trial, it was initially studied as a monotherapy in 32 patients, including those who had received chemotherapy prior to treatment and those without treatment PD-L1-positive TNBC [46]. This showed a positive overall response rate (ORR) of 18.5 percent, so it was given the first ICI approval in TNBC. The ORR in another phase II KEYNOTE-086 study was not as good, showing only 5.3 percent in 170 patients with a PD-L1-unselected pre-treated cancer. In the same study, the ORR was 21.4 percent in 84 without treatment patients, indicating that ICIs are more effective in the first-line metastatic scenario [47]. The KEYNOTE-119 trial, which compared single-agent pembrolizumab to single-agent chemotherapy in pre-treatment metastatic TNBC, found similar results to KEYNOTE-086 in terms of progression-free survival (PFS) and overall survival (OS) [48].

PD-L1 Inhibitors

Atezolizumab/Avelumab: It reverses T cell suppression by directly attacking PD-L1 to inhibit association with the PD-1 and B7-1 receptors [49]. In a phase 1 trial, atezolizumab produced a 10 percent ORR in 115 pre-treated patients, in contrast, patients with PD-L1 negative showed no improvement [50]. In 58 patients who had received significant pre-treatment, the phase 1b JAVELIN trial that evaluated atezolizumab as a monotherapy reported that the ORR was only 5.2 percent [51]. These studies demonstrate the ineffectiveness of a single agent in treating metastatic TNBC, with especially low response rates in the metastatic disease groups that had already received treatment.

Chemotherapeutic Agents Used in Combination With Immunotherapy

Anthracyclines, taxanes, platinum compounds, and a combination of doxorubicin and cyclophosphamide or docetaxel and cyclophosphamide are most commonly used as chemotherapy agents. These agents have shown improved pathologic complete response rates when given along with conventional chemotherapy [52,53]. Chemotherapy in the neoadjuvant context for early-stage TNBC is recommended by several guidelines [54]. The most typical neoadjuvant method is to administer anthracycline and taxane-based chemotherapy sequentially, with the addition of carboplatin, which has been shown to improve the PCR rate [55]. ICI monotherapy was generally effective against metastatic TNBC when the illness was contained; however, in women with progressive disease and an increased metastatic tumor burden, there was little to no response. As a result, researchers began to focus on ICI combined with chemotherapy. Chemotherapy was thought to increase anticancer responses to ICI. Taxanes have the ability to activate toll-like receptors and increase dendritic-cell activity in particular [56]. In patients with TNBC expressing PD-L1, the KEYNOTE-355 study found that first-line treatment with pembrolizumab significantly improved progression-free survival compared to chemotherapy [57].

Patients with stage II/III cancer who received combined chemotherapy and pembrolizumab had a response rate approximately three times higher than those who received chemotherapy alone in the I-SPY 2 study [46]. Pembrolizumab with chemotherapy enhanced response rates (51.2 percent to 64.8 percent) and 18-month event-free survival (EFS) (85.3 percent to 91.3 percent) in both the neoadjuvant and adjuvant contexts in the KEYNOTE-522 study [58,59]. In contrast to the findings of the previous studies, the NeoTRIPaPDl1 research found that conventional chemotherapy in conjunction with atezolizumab did not affect response rates in patients with progressed TNBC [60].

Phase 3 Randomized Controlled Trials

1. KEYNOTE-119/NCT02555657 (completed): Pembrolizumab and chemotherapy were compared in this phase 3 trial of patients with metastatic TNBC. The 1098 patients who were enrolled in the experiment received either pembrolizumab or chemotherapy at random. The primary goals were OS in PD-L1 positive patients and in all patients. With pembrolizumab, the median OS for PD-L1 positive patients was 12.7 months, compared to 11.6 months with chemotherapy (0.057). In the entire population, the median OS for the pembrolizumab group was 9.9 months, while it was 10.8 months for the chemotherapy group (non-significant). The most frequent side effects were neutropenia and anemia [48].

2. KEYNOTE-355/NCT02819518 (active, not recruiting): In the study of *Pembrolizumab (MK-3475) Plus Chemotherapy vs. Placebo Plus Chemotherapy for Previously Untreated Locally Recurrent Inoperable or Metastatic Triple Negative Breast Cancer,* 1372 patients were randomly assigned to one of two therapy groups. In patients with PD-L1 positivity, PFS was 9.7 months in the pembrolizumab group and 5.6 months in the placebo group (p = 0.0012). For all patients, the median PFS was 7.5 months, as opposed to 5.6 months for the control group. The effects of pembrolizumab treatment were enhanced by PD-L1 enrichment. Sixty-eight percent of pembrolizumab patients and 67 percent of placebo individuals [61] reported adverse events.

3. KEYNOTE-522/NCT03036488 (active, not recruiting): The trial enrolled 602 patients who were randomly assigned to one of two therapy groups, that is, pembrolizumab (MK-3475) with chemotherapy versus placebo with chemotherapy as neoadjuvant therapy and pembrolizumab versus placebo as adjuvant therapy and received the primary PCR at the time of surgery as well as EFS. The pembrolizumab group had a 64.8 percent PCR compared to 51.2 percent in the placebo group. The two groups had similar adverse effects. Unlike metastatic trials, the PCR rate improved when compared to chemotherapy alone, regardless of PD-L1 levels; nevertheless, the PD-L1+ group had the highest absolute PCR of 81.7 percent [62].

4. KEYNOTE-242/NCT02954874 (active, not recruiting): This is a phase 3 trial that evaluates pembrolizumab versus a placebo in patients with 1 cm of residual invasive carcinoma and positive lymph nodes after receiving adjuvant neoadjuvant therapy for a year. OS and disease-free survival (DFS) are the most crucial results [62].

5. Impassion130/NCT02425891 (completed): In individuals with untreated metastatic TNBC, a phase 3 trial examined atezolizumab in combination with nab-paclitaxel or a placebo. The trial was randomized and had 451 patients in each group. The treatment was continued until the disease progressed or harmful effects reached an unacceptable level. PFS and OS were the main objectives. Within the two groups, the PFS with atezolizumab was 7.2 months compared to 5.5 months with placebo, and the median OS was 21.3 months compared to 17.6 months. Overall, 15.9 percent of patients who got atezolizumab and 8.2 percent of those who received a placebo experienced adverse events that caused them to stop taking the drug [59].

6. Impassion030/NCT03498716 (recruiting): In *A Study Comparing Atezolizumab (Anti PD-L1 Antibody) in Combination with Adjuvant Anthracycline/Taxane-Based Chemotherapy Versus Chemotherapy Alone in Patients with Operable Triple-Negative Breast Cancer,* another phase 3 trial is now recruiting 2300 people with operable stage II or III TNBC. They were divided into two groups based on surgery type, lymph node status, and PD-L1 status. The major endpoint is DFS [63]. This trial is still recruiting participants, and no findings have been disclosed.

7. Impassion031/NCT03197935 (active, not recruiting): It is a phase 3 trial, with the main goals being PCR in all patients and PD-L1 positive patients. Three hundred and thirty-three patients were randomly randomized to receive atezolizumab with chemotherapy or placebo plus chemotherapy. When compared to the placebo group, the PCR with atezolizumab was 58 percent in all patients as opposed to 41 percent. When it came to PD-L1 positive patients, the difference was even more pronounced, with 69 percent of atezolizumab patients attaining a PCR as opposed to 49 percent in the placebo group [64].

8. NSABP B-59/GBG 96-GeparDouze/NCT03281954 (active, not recruiting): In the study *Clinical Trial of Neoadjuvant Chemotherapy with Atezolizumab or Placebo in Patients with Triple-Negative Breast Cancer Followed After Surgery by Atezolizumab or Placebo,* the NSABP B-59 trial compares neoadjuvant chemotherapy with atezolizumab or placebo to adjuvant mepolizumab or placebo in individuals with early-stage TNBC. The main goals are EFS and PCR in the breast and lymph nodes. No results have been released yet [65].

9. NCT01042379 (recruiting): In the study *I-SPY TRIAL: Neoadjuvant and Personalized Adaptive Novel Agents to Treat Breast Cancer,* the effectiveness of traditional chemotherapy alone and new medications combined with it are compared. The objective is to find better treatment plans for subsets depending on the molecular features (biomarker signatures) of the tumor. It is a multicenter, open-label, randomized phase 2 platform experiment that is still running. The trial is still actively recruiting participants, and no findings have been made public [66].

Other Immunotherapy Strategies

Breast cancer (BRCA) gene-positive tumors have DNA repair deficiencies and so this PARP (poly (ADP-ribose) polymerase) is a nuclear enzyme that aids in the repair of DNA single-strand breaks and is found in over 90 percent of TNBC [67]. PARPi, which target these recombination repair mechanisms and are beneficial in the treatment of breast cancer susceptibility gene 1 (BRCA1) and breast cancer susceptibility gene 2 (BRCA2) mutation carriers with TNBC, are being combined with immune checkpoint inhibition to elicit a stronger antitumor immune response. The release of tumor antigens by PARPi-induced cell death activates invading T cells (NCT03281954) [66,68]. Ongoing trials are investigating the use of PARPi in the treatment of TNBC that are currently recruiting participants.

Another cutting-edge approach in cancer immunotherapy is the use of cancer vaccines, which may trigger an immune response against tumor-specific and tumor-associated antigens. Additionally, it is acknowledged that using immunotherapy as the initial line of metastatic treatment will increase OS rates [69].

Limitations

This study may have some limitations, as the quality of this review depends on available studies and published systematic and literature reviews. Several unpublished study trials are ongoing, recruiting patients for in-depth research on therapeutic strategies and it is not yet known if they will be used in the population. In addition, there may be bias due to study heterogeneity. With these drawbacks, we have made an effort to increase the validity of literature reviews by combining the findings of the many research that have been conducted so far. On the other hand, a thorough investigation would probably result in a stronger connection, a more conclusive result, improved patient treatment, and increased awareness.

## Conclusions

Studies show that PD-1 inhibitors like pembrolizumab used as monotherapy with prior chemotherapy showed an ORR of 18.5 percent and proved to be one of the most effective therapy. On the contrary, using atezolizumab as monotherapy was not very effective in subsequent trials. Also, taxane-based chemotherapy is proven to be the most efficient in terms of PCR rate. Antibodies targeting PD-1/PD-L1/CTLA-4 are mainly focused in combination with chemotherapy to suppress tumor progression. A combination of pembrolizumab and chemotherapy showed a response rate three times higher than using chemotherapy alone. Various phase 3 randomized control trials also depict a combination of PD-1/PD-L1 inhibitors with chemotherapy drugs showing effective response against TNBC. The tumor microenvironment is responsible for the progression, development, and metastasis of tumors, and hence, in-depth research studies of TME and various other drug combinations have to keep going for knowing advancements in treating TNBC. The nature of TNBC is heterogenous and so the combination of various drugs should be continuously tried, and its efficacy should be known through various clinical trials.
